# Supply of Antioxidants vs. Recruit Firefighters’ Cellular Immune Status: A Randomized Double-Blinded Placebo-Controlled Parallel-Group Trial

**DOI:** 10.3390/life12060813

**Published:** 2022-05-30

**Authors:** José Augusto Rodrigues Santos, Tiago Azenha Rama, Domingos José Lopes da Silva, Ricardo J. Fernandes, Rodrigo Zacca

**Affiliations:** 1 Centre of Research, Education, Innovation and Intervention in Sport (CIFI2D), Faculty of Sport (FADEUP), University of Porto, 4200-450 Porto, Portugal; jaugusto@fade.up.pt (J.A.R.S.); ricfer@fade.up.pt (R.J.F.); 2 Service of Immunoallergology, University Hospital Center of São João, 4200-319 Porto, Portugal; tarama@med.up.pt; 3 Service of Basic and Clinical Immunology, Faculty of Medicine, University of Porto, 4200-319 Porto, Portugal; 4 Research Centre in Mathematics and Applications, University of Évora, 7004-516 Évora, Portugal; domingosjlsilva@gmail.com; 5 Porto Biomechanics Laboratory (LABIOMEP-UP), University of Porto, 4200-450 Porto, Portugal; 6 Research Center in Physical Activity, Health and Leisure (CIAFEL), Faculty of Sports (FADEUP), University of Porto, 4200-450 Porto, Portugal; 7 Laboratory for Integrative and Translational Research in Population Health (ITR), 4050-290 Porto, Portugal

**Keywords:** exercise, health, nutrition, exercise physiology, leukocytes, lymphocytes, lymphocyte subsets, firefighters, recruits, training and testing, antioxidants, micronutrients, supplementation

## Abstract

Background: Physical exercise can affect the immune system. We studied the effect of antioxidants on hematological and immune biomarkers after heavy training. Methods: 24 well-trained and well-fed male firefighters were randomly divided into supplemented and placebo groups, and tested for immunology-related variables using venous blood samples in the fasting state, pre- (M1) and post- (M2) five weeks of daily micronutrient supplementation (15 mg of beta-carotene, 200 mg of vitamin C, 136 mg of vitamin E, 200 μg of selenium, 15 mg of zinc, 100 mg of magnesium). Total leukocytes and a differential count for five populations were determined using standard procedures (MAXM—Beckman Coulter Diagnostics; Brea, CA, USA). Lymphocyte subsets were determined through immunophenotyping. Results: Although all values were within the normal range for healthy adults and athletes in the supplemented group (SG), mean CD3^+^CD8^+^, CD8^+^ and CD16^+^CD56^+^ decreased (*p* < 0.05; small to moderate effects), while mean CD4^+^, CD19^+^ and CD4^+^/CD8^+^ increased (*p* < 0.05; small effects) after five-weeks. Regarding the placebo group (PG), higher total leukocyte count (*p* < 0.05; trivial effect) and natural killer cells percentage (CD16^+^CD56^+^; *p* < 0.05; moderate effect) were observed when comparing M1 and M2. Conclusions: Antioxidants supplementation did not alter well-fed male firefighters recruit firefighters’ immune cell response during the five-week physical training program.

## 1. Introduction

Intense physical exercise increases energy breakdown and the formation of oxygen-free radicals [1]. These reactive oxygen species (ROS) have quite a lot of important physiological functions (e.g., cellular signaling, regulation of vascular tone, bactericidal and bacteriostatic activity) [2] but can exacerbate inflammatory response after exercise [3]. ROS can interact with chromatin and make the DNA highly immunogenic [4]. The immune response to oxidative stress can be mitigated through endogenous and exogenous antioxidants [5]. While moderate physical exercise is an ideal stimulus to improve the immune system and antioxidant mechanisms, regular intense physical exercise can accentuate oxidative stress related to immune system dysfunction, inducing a rise in inflammatory markers [6]. The recurrent use of antioxidants is a widespread practice in sports and other intense physical activity situations. For instance, firefighters are exposed to environmental stressors such as heat, smoke, and chemical waste, plus intense physical effort. The pursuit of nutritional resources to reduce some metabolic effects of the imposed environmental and physical activity demand is comprehensible. Likewise, it is well recognized that physical effort can affect the immune system, which mainly depends on the duration and intensity of the stimuli. Prolonged exercise and heavy physical training are associated with depressed immune cell function [7].

Over the last 30 years, exercise-induced effects on circulating immune cells have been the theme of some studies [7,8,9,10,11]. Regular training seems to induce some immune changes that have controversial significance for the athlete’s immune status. Well-trained cyclists and non-trained controls presented immune likeness at the beginning of the training season [12]. After six months of intensive training, cyclists showed, in relation to controls, lower cell counts of leukocytes, lymphocytes, CD3^+^ and CD3^+^CD4^+^ cells, and a lower CD4^+^/CD8^+^ ratio [12]. Other authors suggested that well-trained athletes compared to untrained controls expressed decreased T cell percentage and increased number and percentage of natural killer (NK) cells [13]. Periods of hard swimming training affect innate immunity by reducing CD56 (dim) and increasing CD56 (bright) NK cell subpopulation [14]. Although acute exercise causes relevant changes in the number and relative distribution of leukocyte and lymphocyte subsets in the peripheral blood, these changes are generally transitory, and resting levels are normally reestablished within 24 h after exercise [9]. Despite that, long-lasting periods of intense training can induce immunodepression that can compromise both health and performance [15].

Nutrition is an important factor for immunocompetence. For instance, several nutrients are directly or indirectly associated with immune function, both in sedentary, physically active, and trained subjects. Omega-3 fatty acids attenuate the inflammatory process mediated by cytokines produced by macrophages [16], neutrophils [17], and T cells [18]. Other nutrients have the same immunomodulatory effect: arginine, glutamine [19], and sulfur-containing amino acids (methionine, cysteine, and taurine). Their protective effect against free radicals has an indirect effect on the immune system [20]. Nutritional deficits can accentuate immune alterations induced by exercise [21], highlighting the importance of nutritional supplementation. In addition, some macronutrient supplementation can improve immune response to exercise in high energy spending sports. Although branched chain amino acids (BCAA) supplementation did not affect performance, skeletal muscle damage, or renal function after a 100-km ultramarathon [22], it seems to be useful for immune regulation during exercise [23]. Consuming carbohydrates during prolonged strenuous exercise attenuates the rise in stress hormones and seems to reduce immunodepression [24]. However, in well-fed individuals, the effects of supplementation on immunity are the object of debate. Immune improvement during arduous training throughout micronutrient supplementation is unclear and depends on the micronutrient status of the athletes. In situations of micronutrient deficiency, immune function is depressed, which can lead to an unbalanced host response [25]. In these situations, micronutrient supplementation can be used to mitigate the impaired immune system. Disease can be attenuated by nutritional supplementation. In certain risk groups (e.g., pregnant women, the elderly, and individuals who go through frequent detrimental diets), micronutrient supplementation can be a solution for nutritional deficiencies [26], avoiding immunosuppression.

In sports, improving immune status through micronutrient supplementation is determined by the athlete´s nutritional condition. It seems that in the presence of micronutrient depletion, the immune status is improved through micronutrient supplementation [27]. Magnesium is required for a variety of functions, including immune function. Intracellular free magnesium concentration in NK cells and CD8 T cells regulates their cytotoxicity [28]. Its deficiency can be related to cellular and humoral immune function [29]. In athletes with balanced magnesium status, magnesium supplementation failed to prevent exercise-associated alterations in immune cell function [30]. Zinc is a cofactor in more than 300 enzymes influencing various organic functions with direct effects on the production, maturation, and function of leucocytes. Its supplementation during training can support changes in immune status in response to exercise training [31]. Zinc supplementation increased TNF-α, IL-2, and IFN-γ in wrestlers [32], though athletes are unlikely to benefit from zinc supplementation during periods of increased training volume [33]. Additionally, zinc supplementation can be deleterious to monocyte function in some conditions [34]. Vitamin C deficiency impairs immunity and increases susceptibility to infections. Vitamin C is stored in phagocytic cells, such as macrophages and neutrophils, acting in microbial killing and enhancing the proliferation and differentiation of B- and T- cells [35]. While vitamin C supplementation can improve immune status and reduce the incidence of upper respiratory tract infection in ultramarathon runners, the same benefits were not stated in non-runner controls [36]. Gleeson et al. [37] postulated that high doses of antioxidant vitamins are unlikely to prevent exercise-induced immunosuppression. Vitamin E is a potent modulator of the immune cells. Although deficiency is rare, it seems that supplementation can boost the immune response to pathogens and reduce the risk of infection [38]. Beta-carotene is a dietary proteinoid enzymatically converted in the gut to retinoid. Carotenoids might help protect immune cells from oxidative damage, thus enhancing their immune function [39,40]. Selenium, a trace mineral found in grains, vegetables, seafood, meat, dairy products, and nuts [41], is linked to immune response through its incorporation into selenoproteins. Selenium supplementation boosts T cell proliferation, NK cell activity, and immune innate cell functions [42]. Current evidence suggests that it may be important to maintain an adequate selenium status [43].

Immune status improvement, when supplemented by a single nutrient, seems not to be as effective as a mixture of several nutritional supplements [44]. Besides, large doses of vitamin C [45] and vitamin E [46] can be deleterious for health and performance. Despite that, trivial changes were observed in serum concentration of humoral immunity biomarkers (C3 and C4 complements) and total hemolytic complement activity (CH100) after five weeks of micronutrient supplementation in male recruit firefighters [47]. Serum immunoglobulins (IgG, IgA, and IgM) were also assessed during the physical training program for male recruit firefighters [48]. Although mean values remained within the reference values, some changes observed after five weeks for IgG and IgM might reflect some immune protection [48]. Therefore, the purpose of this investigation was to determine if an antioxidant supplementation could improve immune status in male recruit firefighters undergoing five weeks of a physical training program. We hypothesize that even in well-fed subjects, leukocytes, lymphocytes, and lymphocyte sub-fractions are altered by antioxidant supplementation during the physical training program.

## 2. Materials and Methods

### 2.1. Study Design

This was a randomized double-blinded and placebo-controlled parallel-group trial, with the supplemented group receiving a branded supplement pill (Ever-Fit Plus^®^, Prisfar, Portugal) over 35 consecutive days. The ingredients of each supplement pill (Ever-Fit Plus^®^ Prisfar, Portugal) were: 15 mg of beta-carotene, 200 mg of vitamin C, 136 mg of vitamin E, 200 μg of selenium, 15 mg of zinc, and 100 mg of magnesium). The subjects were informed of the risks associated with their participation, and informed consent was obtained from all subjects involved in the study.

### 2.2. Participants

**Figure 1:**
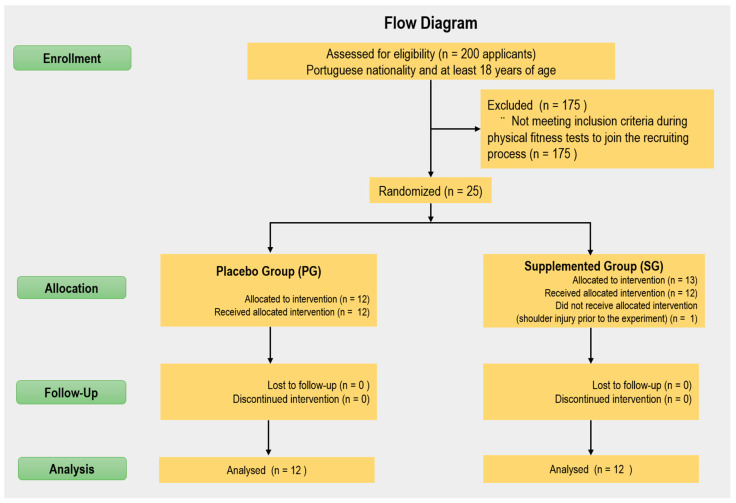
Flow diagram of the study.

The participants were chosen from an initial population of ~200 applicants of Portuguese nationality and at least 18 years of age, who were selected through physical fitness tests. The inclusion criteria were that subjects should be professional recruit firefighters; healthy (assessed through medical tests); with no muscular, bone, or articular pathologies, without visual or hearing deficits; and classified in physical conditioning tests. Participants with any physical or organic pathology were excluded. Then, the top 25 joined the recruiting process. However, one firefighter recruit was excluded due to a shoulder injury prior to the experiment. Thus, 24 newly enlisted male, well-trained and well-fed recruit firefighters belonging to the Firefighters Sapper’s Battalion (Porto, Portugal) were randomly divided (concealed allocation was implemented) into supplemented group (SG) (n = 12) and placebo group (PG) (n = 12) (Figure 1) [49]. The researchers and participants were blinded to the group assignment. Both groups were evaluated at baseline (M1) and five weeks later (M2). The study protocol has already been published by our research group [47,48].

Age, anthropometrical characteristics, and toxic habits of participants are shown in Table 1.

### 2.3. Procedures

#### 2.3.1. Training Program

**Figure 2:**
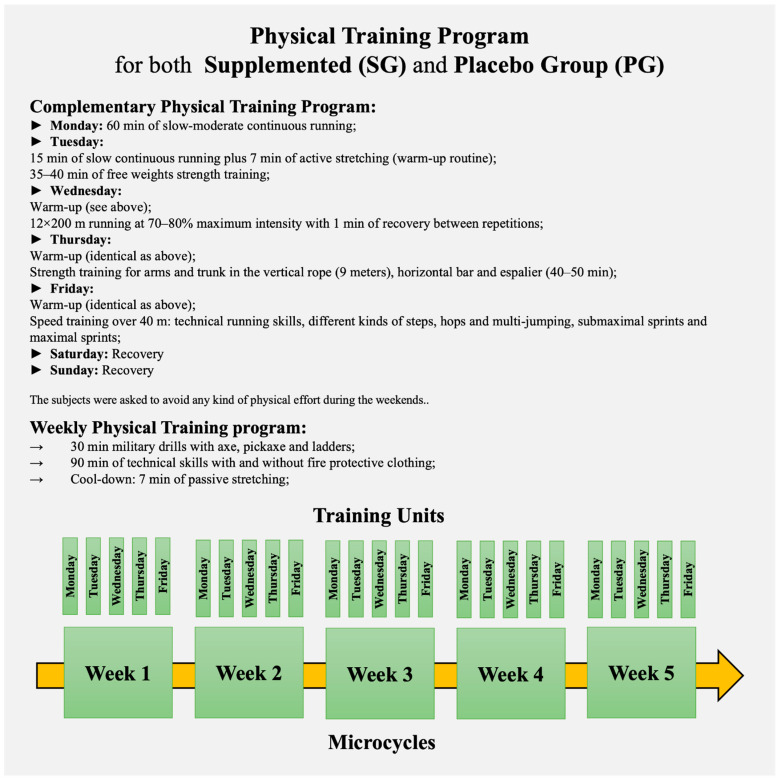
Physical Training Program.

The training period included 5 microcycles (weeks) of 5 training units (plus 30 min of military drills with axe, pickaxe, and ladders, and 90 min of technical skills with and without fire protective clothing) and 2 resting days (Figure 2).

#### 2.3.2. Body Mass Assessment

Anthropometrical assessments included the measurement of stature, mass, and skinfold thickness at triceps, biceps, sub-scapular and supra-iliac sites. Skinfold thicknesses were measured with a Harpenden skinfold caliper (Baty International, Burgess Hill, UK; http://www.harpenden-skinfold.com/, accessed on 1 May 2022). Body fat mass was estimated from the sum of these skinfolds using the Durnin & Womersley (1974) formulae [50] for body density calculation, and the Siri equation [51] determined body fat percentage.

#### 2.3.3. Physical Fitness Level

All participants received the same physical training and professional skills program over the 3 months prior to the experiment. Physical fitness in-between groups were similar at the beginning of the study (Table 2).

#### 2.3.4. Nutritional Assessment

Dietary intake was assessed on 2 weekdays and 1 weekend day (one week prior to intervention) using a photo album with 134 pictures containing average raw/cooked food portions. Mean daily food intake was converted to nutrients estimates by using the ESHA’s Food Processor Nutrition Analysis software [52]. Dietary intake values prior to the experiment were similar between groups (Table 3). Since both groups presented similar nutritional intake before intervention and firefighters had the same meals during the intervention, they were not tested again for these variables.

#### 2.3.5. Supplementation and Placebo

A pill with a supplement (Ever-Fit Plus^®^ Prisfar, Porto, Portugal) or placebo was given to the respective groups for 35 consecutive days. The placebo group received a placebo pill with a powder of maltodextrin, artificial flavor, and color over the same period.

#### 2.3.6. Blood Sampling

The first blood drawn was performed three months after the beginning of the recruit (M1) and the second (M2) 35 days later. After two resting days, venous blood samples (5 mL) drawn from the antecubital vein after overnight fasting were collected into vacutainers containing ethylenediaminetetraacetic acid (EDTA) and processed within 6 h. Laboratory procedures were performed in the Immunology Service of Centro Hospitalar Universitário de São João, Porto, Portugal.

#### 2.3.7. Immunophenotyping

Total leukocytes and differential count for five populations were done using standard procedures (MAXM— Beckman Coulter Diagnostics; Brea, CA, USA).

#### 2.3.8. Monoclonal Antibodies

Mouse monoclonal antibodies were used and directed against leukocyte cell surface antigen and conjugated to fluorescein isothiocyanate (FITC) or phycoerythrin (PE). Cluster, clone, fluorescent stain, origin, and antibody specificity are summarized in Table 4.

#### 2.3.9. Samples Preparation for Flow Cytometry

Lymphocyte subsets were determined through direct immunofluorescence. EDTA-treated blood was incubated (20 min, 4 °C, dark) with 15 μL of monoclonal antibodies conjugated with FITC or PE. Subsequently, erythrocytes were lysed for 10 min with 2 mL of FACS Lysing Solution^®^ (Becton Dickinson, San José, CA, USA). Finally, the cells were washed in PBS, centrifuged at 1500 r∙min^−1,^ and maintained again in PBS. List mode files were acquired within 2 h in a flow cytometer (FACScan, BD).

#### 2.3.10. Data Collection and Analysis

FACScan Lysis II 1.1. (Becton Dickinson, San José, CA, USA) was used to draft graphics of points for lymphocyte separation using frontal dispersion windows (FSC) vs. lateral dispersion windows (SSC) and confirmed by the gate in CD45 vs. SSC, with windows in the cells with the highest fluorescence intensity in CD45. One thousand events were analyzed. For the multipliers, FL1, and FL2, 600 and 581 volts were applied, respectively, with a linear amplification for FL1 and a spectral compensation for FL2. For confirmation of the analyzed population, the lymphocyte percentage obtained was compared with the different values of the 5 subpopulations, and the result was obtained with the first graphic of points−FL1 versus SSC−using CD45/CD14 (Leucogate-BD). Cells showing double mean intensity of fluorescence compared with the negative control were considered positive.

### 2.4. Statistical Analyses

The statistical analysis and reporting of this study were conducted in accordance with the consolidated standards of reporting trials (CONSORT) guidelines [53], with the primary analysis based on the full analysis set. The sample size, estimated with G * Power 3.1.9.6 (Heinrich Heine Universität Düsseldorf, Düsseldorf, Germany), was deemed adequate assuming an effect size of 0.65, 85% of statistical power, and 0.05 α error probability. Shapiro-Wilk test showed the normality of the distribution for all variables. After checking the equality of variance, we applied an independent-samples *t*-test to compare groups. Paired-samples *t*-test was applied to compare before (M1) and after (M2) supplementation. Effect sizes (Cohen’s *d*) were interpreted with the following criteria: 0–0.19 trivial, 0.2–0.59 small, 0.6–1.19 moderate, 1.2–1.99 large, 2.0–3.99 very large, and >4.0 nearly perfect. All statistical analysis was performed using SPSS (Statistical Package for the Social Sciences), v.28.0. Statistical significance was set as α = 5%.

## 3. Results

Table 5 shows the changes noted in the supplemented group. Several immune indicators suffered important changes, despite that all values were within the normal range found for healthy adults and athletes. Differences are due to the higher mean values of M1 in CD3^+^CD8^+^ (small effect), CD8^+^ (small effect) and CD16^+^CD56^+^ (moderate effect) variables, while in CD4^+^, CD19^+^ and CD4^+^/CD8^+^ variables, the M2 showed higher mean values (small effects).

Table 6 expresses the placebo group experiment alterations. All values were within the normal range. Total leukocyte count and natural killer cells (CD16^+^CD56^+^) percentage suffered moderate and trivial increase, respectively.

Table 7 shows that groups were similar for all biomarkers in both evaluation moments (M1 and M2). Although the intra-group variations were significant for some indicators, there were no differences between the groups in both evaluation moments.

Body mass and body composition between groups remained similar after 5-weeks (Table 8). Trivial changes (not significant) in body mass and percentage of body fat were observed after this period.

## 4. Discussion

Exercise is an important modulator of the immune system and affects both innate and adaptive immune responses. Innate immune response is triggered by any type of pathogen. It reacts through chemotactic, phagocyting, cytokines-secreting, and cell-killing quick pathways, while the adaptive immune system responds more slowly by activating dendritic and T and B cells [54]. “Trained immunity”, which represents the concept of long-term adaptation of innate immune cells [55], effectively eliminates invading pathogens.

Exercise, which can affect the immune system in different ways, is conditioned by several factors, among which the training level and the nutritional intake profile of the subjects stand out [56]. However, our study, which was undertaken to assess the influence of an antioxidant supplement on immune status in subjects undergoing a physical training program, showed trivial changes in the selected immune variables. Leukocyte and lymphocyte counts, as well as lymphocyte percentages, were similar for supplemented (SG) and placebo group (PG) at both moments of evaluation. Mean values for both groups were within laboratory references. Regarding the first moment of evaluation (M1), leukocyte counts for both groups were lower than the mean values found by Nieman et al. [57] for marathoners (5.23 ± 0.27∙10^9^∙L^−1^) and controls (6.05 ± 0.38∙10^9^∙L^−1^). Regarding the second moment (M2), leukocyte count showed a slight but statistically significant increase in PB. Even though intense physical efforts may induce a transitory leukocytosis [58], these changes are most likely spurious, as resting leukocyte count is not different when comparing active and non-active subjects [59] and may fluctuate during periods of hard training [60], namely in the 24 h following heavy physical exertion [61,62]. Resting leukocyte count decreased progressively after intensified endurance training in some studies [63,64], somehow contrasting with our data, despite similar values (M1 vs. M2) after supplementation. Diment et al. [63] observed that a nutritional supplement attenuated the decrease in leukocyte count (observed in control group) subsequent to a hard training period. The loss of body mass induced by energy deficit verified in the study of Diment et al. [63] and the time lapse between the end of the exercise and the blood draw (24 vs. 48 h) may explain the discrepancy between both studies.

Absolute [*n*∙(mm^3^)^−1^] and relative (%) lymphocyte counts were within normal laboratory range and were similar for both groups in the two moments of evaluation. Contrary to our findings, after periods of arduous training, a significant decrease in lymphocyte count was verified [63]. Highly active subjects can have abnormally low levels of lymphocytes [65] which was not verified in our study. Eventual reasons for this discrepancy could be: (i) the diversity of the training program, which integrates different exercise modes not overloading a single metabolic system (e.g., endurance, strength/power, speed, and flexibility); (ii) the good physical conditioning of our subjects, (iii) the recovery period after each microcycle was sufficient to return potential immune changes induced by weekly training to baseline levels, or (iv) the physical loads were not sufficiently intensive to promote significant immune alterations.

Mean lymphocyte values for both groups in our study were higher than the lower normal laboratory reference [1500∙(mm^3^)^−1^]. When diet is adequate, the immune response to strenuous exercise is attenuated [66], and lymphocyte concentration after exertion is improved [63].

Although carbohydrate intake in our subjects was lower than the recommendations for very active people, it seems that the diet was balanced enough to match the energetic demands of the training program. In our study, no immune signals of unregulated inflammation (e.g., a dramatic increase of leukocytes and reduction of lymphocytes) were verified, which highlights diet adequacy and/or recovery efficacy. An adequate diet can boost immunocompetence and reduce inflammatory processes [67]. Micronutrient supplementation can improve the immune system only in situations of nutritional deficiency [68].

Lymphocyte subfractions analysis provides a picture. Although the differences between groups were not significant in any moment of evaluation, the behavior of each group was different. In the supplemented group CD4^+^ cell count increased (*p* < 0.05) while CD8^+^ cell count decreased (*p* < 0.01). In the placebo group, changes were only significant for NK cells (CD16^+^CD56^+^). We can postulate that the differences verified, although significant, are circumstantial and not related to nutritional treatment. Only situations of hard training conjugated with an unbalanced diet can significantly alter immune status. Trushina et al. [69], in weightlifters undertaking intense physical training under an inadequate macronutrient intake, verified significant alterations of T cells subsets (marked increasing of CD8^+^ T cells and decreasing of CD4^+^ T cells). An evident increase of CD8^+^ cells with a concomitant decrease of CD4^+^ cells can signify immunodeficiency and subclinical infection [70].

The lymphocyte subsets changes verified in the SG brought them closer to the PG in the second moment of evaluation. Nieman et al. [57] observed that NK cells and T cells in marathoners were similar to sedentary controls, reinforcing the similarity of lymphocyte subsets between groups. Both groups in our study were identically active and seemed immunologically similar, albeit in the supplementation.

Periods of hard training can decrease absolute counts of circulating NK cells [12,14]. NK cells decreased in SG (*p* < 0.05) while remaining stable in PG. In both moments of evaluation, relative NK cell counts were higher than the values found by Baj et al. [12] in cyclists. Thus, we cannot give high immunological significance to the changes found in our study. The effect of supplementation on NK cell concentration seems to be dependent on the supplement type and type of exertion. While blueberries, rich in vitamins and phytochemicals, improve immunity, increasing NK cell levels after long-lasting intense running [67], ginseng does not affect the immune response to moderate exercise [71]. It was also reported that acute multi-nutrient supplement ingestion (1000 mg quercetin, 120 mg epigallocatechin 3-gallate, 400 mg isoquercetin, 400 mg each eicosapentaenoic acid [EPA], and docosahexaenoic acid [DHA], 1000 mg vitamin C, and 40 mg niacinamide), 15 min before heavy exertion caused a strong increase in plasma quercetin levels but did not counter post-exercise inflammation or immune changes relative to placebo [72]. When moderate training induces a loss of body mass and fat, resting NK cells count increases by 21% [73]. In our subjects, body mass and fat mass remained stable during the study, so the slight changes verified cannot be related to body composition alterations.

CD19^+^ cells are acutely altered by strenuous exercise [11], returning to basal values within 24 h [7]. It was shown that systematic exercise chronically increased B cell blood counts in rats [74]. In our study, B cells increased significantly in SG while PG decreased. As both changes were within the reference range values for healthy individuals and athletes [75,76,77], we are led to believe that the changes are not linked to supplementation. Chronic changes in circulating lymphocyte counts induced by training seem negligible [9]. Only when training volume is sufficient to induce a negative energy balance are CD19^+^ cell counts significantly reduced [73]. However, even in elite female gymnasts with signals of malnutrition, CD19^+^ cell counts are similar to controls with healthier nutritional behavior [78].

T lymphocytes (T cells) are central for both innate and acquired immune systems by producing specific pro- and anti-inflammatory cytokines [79,80]. During high-intensity exercise, peripheral blood counts of CD4^+^ and CD8^+^ T cells rise with a larger relative increase in CD8^+^ T cells due to their higher density of beta-2 adrenoceptors [78]. Usually, T cells return to baseline values 2–3 h post-exercise [81].

For PG, CD4^+,^ and CD8^+^, T cell counts are almost identical between moments. For S, CD4^+^ increased while CD8^+^ decreased, both reaching statistical significance. However, the differences between groups were not statistically significant at any moment of evaluation, which removes immunological meaning from the verified changes. Other studies point to the same outcomes. In elite Australian swimmers, there were no significant changes in numbers or percentages of B or T cell subsets during the season [82].

CD4^+^/CD8^+^ ratio was significantly reduced (−13.6%) after repeated bursts of prolonged exercise, but ten days of passive rest are sufficient to recover and exceed basal levels [83]. In trained subjects, 6-h after exertion, a significant rise in CD4^+^/CD8^+^ ratio was verified but returned to basal level in 24 h [84]. In this study, SG increased significantly CD4^+^/CD8^+^ ratio while PG showed a slight but non-significant decline. The differences between groups were not statistically significant at any moment of evaluation. Contrasting with our findings, several authors verified that systematic training tended to reduce the CD4^+^/CD8^+^ ratio [58,85,86]. However, some previously reported data support our results. In the study by Shore et al. [73], for 12 weeks, in addition to a control group, two groups were randomly assigned to aerobic exercise (70–85% of maximal heart rate) three sessions∙week^−1^ and 4–5 sessions∙week^−1^. Contrary to the other groups, the group which trained 4–5 times∙week^−1^ experienced a negative energy balance (expressed by a significant reduction in body mass and body fat percentage). None of the groups showed significant changes in the CD4^+^/CD8^+^ ratio.

Different training protocols and different nutrition statuses can be reasons for these discrepancies. In our study, the increase verified in SG could be attributed to supplementation. However, as the relatively higher value of PG was maintained in the second moment of evaluation, it is difficult to sustain the belief that supplementation was the cause for the change in SG. Changes verified for the CD4^+^/CD8^+^ ratio are in line with the behavior of the other selected immune markers and did not point out any signal of immunodeficiency or immune improvement.

It is important to acknowledge some shortcomings and potential limitations of our study. Although firefighters were well-fed, antioxidant absorption in the organism is modified by several aspects, and it would be appropriate to know their basal plasma or serum antioxidant concentrations. Although participants reported avoiding physical efforts over the weekends during the study period, it was not monitored. The kinetics of the variable’s response cannot be entirely observed when only pre- and post-exercise blood samples are compared. Body composition estimation through DEXA would be more accurate, but we could not access this device. Finally, the comparison between firefighters and other exercise modalities (e.g., marathon runners) might be appropriate. Typically, the training intensity for marathon runners is mainly set at the ventilatory threshold during the initial weeks of the training program, moving progressively toward a “between thresholds to marathon pace” intensity [87]. In fact, there are few severe-extreme intense training workouts per microcycle (week), and the remaining content is developed at lower intensities (according to the training level of each runner) [87,88]. Typically, the weekly training workload of firefighter recruits (as those from our study) is higher than marathon runners, being composed of intense physical efforts in their daily routine (see Figure 2), e.g., the ascent-descent to the school-house with a partner on one’s back or rescue exercises in toxic smoke conditions with and without protection). It implies increased acute stress on the immune system, which according to our study does not have chronic immunological implications. As far as we know, there is no study assessing immunological changes in firefighters close to ecological conditions as in the current study. Thus, we searched for studies verifying the effect of supplementation (positive, negative, or neutral) in other experimental designs.

## 5. Conclusions

The supply of vitamins and minerals supplementation with Ever-Fit Plus^®^ did not alter well-trained and well-fed recruit firefighters’ immune cell response during the five-week physical training program. Absolute leukocyte counts showed a trend to increase with the continuity of training, while absolute and relative lymphocyte counts remained stable between the two evaluation moments. Although there were some significant intra-group changes in lymphocyte subsets, no significant differences were found between groups. The magnitude of immune changes in both groups, clearly within normal reference values, does not allow for establishing any link between supplementation and the alterations verified.

## Figures and Tables

**Table 1:** Age, toxic habits, and anthropometrical characteristics of the participants.

Variables	Supplemented Group (SG) n = 12	Placebo Group (PG) n = 12
**Age** (years) **Height** (cm) **Body mass** (kg) **Fat mass** (% body mass)	22.1 ± 1.9 175 ± 4 68.9 ± 7.4 9.6 ± 2.0	23.9 ± 0.3 174 ± 4 69.3 ± 12.3 10.2 ± 2.0
**Smoking** (n) **Alcohol drinking** (n)	3 10	7 9

Data for age and anthropometrical characteristics are shown as mean and respective standard deviation. The differences between groups are not statistically significant (*p* > 0.05).

**Table 2:** Results of physical fitness tests at baseline (mean ± SD) for both groups.

Indicators		Supplemented Group (SG) n = 12	Placebo Group (PG) n = 12
	**Bench Press with 50 kg** (reps)	12 ± 7	14 ± 6
	**Chin-ups** (reps)	16 ± 1	16 ± 2
	**Sprint 50-m** (s)	7.0 ± 0.0	7.0 ± 0.1
	**Cooper Test** (m)	3007 ± 127	3120 ± 106
**WINGATE TEST**	**Relative Peak Power Output** (watt/kg)	10.5 ± 0.5	10.8 ± 0.1
	**Relative Mean Power Output** (watt/kg)	7.8 ± 0.5	8.1 ± 0.3
	**Relative Minimum Power Output** (watt/kg)	5.7 ± 0.8	5.8 ± 0.6
	**Fatigue Index** (%)	45.9 ± 6.4	45.2 ± 6.8
	**Squat Jump** (cm)	37.3 ± 5.7	37.8 ± 3.5
	**Counter Movement Jump** (cm)	38.3 ± 6.2	39.4 ± 5.8

Differences between groups were not statistically significant (*p* > 0.05).

**Table 3:** Nutritional intake at baseline (mean ± SD).

Variables	Supplemented Group (SG) n = 12	Placebo Group (PG) n = 12
**Energy intake** (kcal)	3050 ± 600	2985 ± 580
**Carbohydrate** (%)	51.2 ± 5.8	50.8 ± 3.1
**Protein** (%)	18.4 ± 2.5	19.2 ± 2.1
**Fat** (%)	30.2 ± 3.3	30.8 ± 2.8
**Vitamin C** (mg)	147.2 ± 58.2	152.8 ± 47.3
**Vitamin E** (mg)	7.9 ± 2.3	8.3 ± 2.8
**Beta-carotene** (μg)	165.1 ± 4.3	158.3 ± 7.2
**Magnesium** (mg)	431.4 ± 76.8	411.9 ± 82.5
**Selenium** (μg)	163.3 ± 22.7	161.9 ± 54.2
**Zinc** (mg)	16.9 ± 5.7	17.1 ± 4.2

Differences between groups were not statistically significant (*p* > 0.05).

**Table 4:** Cluster, clone, fluorescent stain, origin, and antibody specificity.

Cluster	Fluorocrom	Clone	Origin	Main Cell Reactivity
**CD3^+^**	FITC	SK 7	Becton **	T cells
**CD4^+^**	PE	SK 3	Beckman Coulter ***	Helper T cells
**CD4^+^CD45RA^+^**	FITC	2H4	Beckman Coulter ***	Helper T naïve cells
**CD8^+^**	PE	SK 1	Becton **	Cytotoxic T cells
**CD57^+^/CD8^+^**	FITC/PE	HNK-1, SK 1	Becton **	Cytotoxic T cells with no MHC restriction
**CD3-CD16^+^&56^+^**	FITC/PE	SK 7, B 73.1, My 31	Becton **	Natural Killer cells
**CD19^+^**	FITC	4 G 7	Becton **	B cells

** Becton Dickinson, San José, Califórnia, USA; *** Beckman Coulter Diagnostics; Brea, California, USA; Isotype controls from Becton and Beckman Coulter were used.

**Table 5:** Immune cell changes (mean ± SD) after 5-weeks for the supplemented group (SG).

Indicators	M1 (Baseline)	M2 (after 5-Weeks)	*p*-Value, Cohen’s *d*
**Leukocytes** (n/mm^3^)	5091.6 ± 956.8	5708.3 ± 1937.4	0.236, −0.40 “small”
**Lymphocytes** (%)	38.2 ± 6.8	37.3 ± 12.0	0.796, 0.09 “trivial”
**Lymphocytes** (n/mm^3^)	1914.4 ± 337.6	2000.1 ± 598.7	0.466, −0.17 “trivial”
**CD3^+^**	72.8 ± 6.6	73.7 ± 7.9	0.445, −0.12 “trivial”
**CD3^+^CD4^+^**	41.3 ± 5.7	43.7 ± 8.0	0.055, −0.34 “small”
**CD57/CD8^+^**	4.4 ± 2.2	3.7 ± 2.6	0.079, 0.29 “small”
**CD3^+^CD8^+^**	27.7 ± 4.9	26.5 ± 4.6	0.046 *, 0.25 “small”
**CD4^+^**	41.8 ± 5.7	44.4 ± 8.1	0.039 *, −0.37 “small”
**CD8^+^**	32.1 ± 4.5	30.3 ± 4.8	0.006 *, 0.38 “small”
**CD19^+^**	10.6 ± 3.0	12.3 ± 3.4	0.023 *, −0.53 “small”
**CD16^+^CD56^+^**	15.9 ± 4.0	12.0 ± 4.8	0.029 *, 0.88 “moderate”
**CD4^+^/CD8^+^**	1.5 ± 0.4	1.7 ± 0.5	0.029 *, −0.44 “small”

Cells with a cluster of differentiation are expressed in percentage of lymphocytes.* Statistically significant differences (*p* < 0.05).

**Table 6:** Immune cell changes (mean ± SD) after 5-weeks for the placebo group (PB).

Indicators	M1 (Baseline)	M2 (after 5-Weeks)	*p*-Value, Cohen’s *d*
**Leukocytes** (n/mm^3^)	5075.0 ± 1037.6	5741.7 ± 992.2	0.010 *, −0.65 “moderate”
**Lymphocytes** (%)	37.5 ± 5.1	36.3 ± 4.5	0.425, 0.24 “small”
**Lymphocytes** (n/mm^3^)	1901.8 ± 461.9	2082.2 ± 436.8	0.119, −0.40 “small”
**CD3^+^**	74.9 ± 8.0	74.6 ± 8.1	0.834, 0.03 “trivial”
**CD3^+^CD4^+^**	43.9 ± 8.7	43.1 ± 7.3	0.489, 0.09 “trivial”
**CD57/CD8^+^**	3.5 ± 2.9	3.6 ± 2.7	0.727, −0.03 “trivial”
**CD3^+^CD8^+^**	26.9 ± 9.8	27.4 ± 9.6	0.165, −0.05 “trivial”
**CD4^+^**	44.4 ± 8.6	43.4 ± 7.3	0.580, 0.12 “trivial”
**CD8^+^**	30.3 ± 8.1	31.0 ± 7.8	0.140, −0.08 “trivial”
**CD19^+^**	12.6 ± 4.4	11.7 ± 4.5	0.072, 0.20 “small”
**CD16^+^CD56^+^**	11.9 ± 6.6	12.8 ± 5.9	0.049 *, −0.14 “trivial”
**CD4^+^/CD8^+^**	1.9 ± 1.2	1.8 ± 1.0	0.076, 0.09 “trivial”

Values are mean ± standard deviation. Cells with a cluster of differentiation are expressed in percentage of lymphocytes. * Statistically significant differences (*p* < 0.05).

**Table 7:** Comparison between groups at baseline and after 5-weeks.

Indicators	SG vs. PG 1st Assessment (M1) (*p*-Value, Cohen’s *d*)	SG vs. PG 2nd Assessment (M2) (*p*-Value, Cohen’s *d*)
**Leukocytes** (n/mm^3^)	0.968, 0.01 “trivial”	0.958, −0.02 “trivial”
**Lymphocytes** (%)	0.761, 0.11 “trivial”	0.787, 0.11 “trivial”
**Lymphocytes** (n/mm^3^)	0.940, 0.03 “trivial”	0.705, −0.15 “trivial”
**CD3^+^**	0.503, −0.28 “small”	0.786, −0.11 “trivial”
**CD3^+^CD4^+^**	0.402, −0.35 “small”	0.864, 0.07 “trivial”
**CD57/CD8^+^**	0.396, 0.34 “small”	0.969, 0.03 “trivial”
**CD3^+^CD8+**	0.795, 0.10 “trivial”	0.791, −0.11 “trivial”
**CD4^+^**	0.384, −0.35 “small”	0.865, 0.12 “trivial”
**CD8^+^**	0.515, 0.27 “small”	0.770, −0.10 “trivial”
**CD19^+^**	0.203, −0.53 “small”	0.735, 0.15 “trivial”
**CD16^+^CD56^+^**	0.086, 0.73 “moderate”	0.723, −0.14 “trivial”
**CD4^+^/CD8^+^**	0.232, −0.44 “small”	0.617, −0.12 “trivial”

Supplemented group (SG) (n = 12). Placebo group (PG) (n = 12).

**Table 8:** Body mass and body composition (mean ± SD) at baseline and after 5-weeks for both groups.

Indicators	Supplemented Group (SG)	Placebo Group (PB)	*p*-Value
**Body mass** (kg):			
M1	68.9 ± 7.4	69.3 ± 12.3	0.439, −0.03 “trivial”
M2	69.2 ± 7.2	69.5 ± 10.8	0.562, −0.03 “trivial”
**Fat mass** (%):			
M1	9.6 ± 2.0	10.2 ± 2.0	0.786, −0.29 “small”
M2	9.7 ± 2.1	10.0 ± 1.9	0.882, −0.14 “trivial”

## Data Availability

The data presented in this study are available on request from the corresponding author.
